# Modified Dixon MRI to detect subclinical inflammation in clinically suspect arthralgia as a risk factor for rheumatoid arthritis development: should we image one or two hands?

**DOI:** 10.1136/rmdopen-2025-006621

**Published:** 2026-03-04

**Authors:** Daniek van der Kaaij, Stijn Claassen, Hanna W van Steenbergen, Edwin H G Oei, Pascal H P de Jong, Monique Reijnierse, Annette H M van der Helm-van Mil

**Affiliations:** 1 Department of Rheumatology, Erasmus Medical Center, Rotterdam, The Netherlands; 2 Department of Radiology and Nuclear Medicine, Erasmus Medical Center, Rotterdam, The Netherlands; 3 Department of Rheumatology, Leiden University Medical Center, Leiden, The Netherlands; 4 Department of Radiology, Leiden University Medical Center, Leiden, The Netherlands

**Keywords:** Arthritis, Rheumatoid, Inflammation, Magnetic Resonance Imaging, Risk Factors

## Abstract

**Objectives:**

MRI-detected subclinical inflammation in clinically suspect arthralgia (CSA) predicts progression to inflammatory arthritis (IA) and rheumatoid arthritis (RA) and is incorporated in EULAR/ACR risk stratification criteria. Conventional MRI is hampered by long scan times, intravenous contrast and high costs, while modified Dixon (mDixon) MRI, with 5 min scan time and no intravenous contrast, is much more feasible. To optimise mDixon MRI utility, we compared bilateral versus unilateral hand analysis for detecting subclinical inflammation. We also studied the distribution of subclinical inflammation in CSA.

**Methods:**

139 patients of the CSA Leiden cohort were included. mDixon MRIs of bilateral wrist and metacarpophalangeal 2–5 joints were scored for subclinical inflammation (synovitis/tenosynovitis/osteitis) using RA MRI scoring. The hand with the most self-reported painful joints was used for unilateral analysis. Patients were followed for ≥6 months, with IA and RA development assessed at 6 months. We compared prognostic performance of bilateral versus unilateral MRI-detected subclinical inflammation.

**Results:**

Subclinical inflammation detected on bilateral MRI was more strongly associated with IA development than on unilateral MRI; 4.80 (95% CI 1.09 to 21.11) versus 2.34 (95% CI 0.84 to 6.49). Bilateral analysis resulted in a 25% higher sensitivity and 15% lower specificity, with a net 10% increase in correctly classified patients. Results for RA development were similar, with HRs of 8.17 (95% CI 1.06 to 62.86) versus 3.23 (95% CI 0.98 to 10.66), and 20% higher sensitivity. Subclinical inflammation was unilateral in 52% of patients and scanning one hand would miss a quarter of patients.

**Conclusion:**

Bilateral MRI of the hands is preferable to unilateral MRI for detecting subclinical inflammation in CSA, because of its asymmetrical distribution.

**WHAT IS ALREADY KNOWN ABOUT THIS TOPIC**

- The presence of MRI-detected subclinical inflammation is a predictor for progression from clinically suspect arthralgia (CSA) to inflammatory arthritis (IA) and rheumatoid arthritis (RA).
- The modified Dixon (mDixon) MRI protocol is fast (5 min scan time), runs on standard 3T MRI scanners and does not require administration of intravenous contrast, unlike conventional contrast-enhanced MRI. This mDixon sequence makes MRI feasible for daily practice in CSA. It is, however, unknown if scanning both hands is preferable to scanning only one hand.

**WHAT THIS STUDY ADDS**

- Half of patients with CSA show asymmetrical subclinical inflammation affecting only one hand.
- mDixon MRI data of two hands resulted in a higher sensitivity for detecting subclinical inflammation and a stronger association with IA and RA development compared with data of one hand.

**HOW THIS STUDY MIGHT AFFECT RESEARCH, PRACTICE OR POLICY**

- Bilateral MRI data is preferable when using the presence of subclinical joint inflammation as a risk factor for IA and RA development in patients with CSA.

## Introduction

 In patients with clinically suspect arthralgia (CSA), the presence of MRI-detected subclinical inflammation is a known predictor for progression to inflammatory arthritis (IA) and rheumatoid arthritis (RA).^1 2^ Therefore, MRI-detected subclinical inflammation, and tenosynovitis in particular, is incorporated in the European Alliance of Associations for Rheumatology (EULAR) / American College of Rheumatology (ACR) risk stratification criteria for RA development, and performing MRI as part of the diagnostic process in CSA has been promoted.^3^ However, contrast-enhanced MRI has several disadvantages that hamper its widespread implementation in clinical practice. MRI is associated with long scan times and requires intravenous contrast to detect synovitis and tenosynovitis, which both contribute to high costs. To overcome these disadvantages, a modified Dixon (mDixon) MRI has recently been proposed: it is fast (5 min scan time) and does not require administration of contrast.^4^,^6^ Importantly, initial results suggest comparable accuracy to contrast-enhanced MRI in detecting subclinical inflammation.^6^ To use the mDixon MRI sequence as optimally as possible, it is important to assess whether one or two hands should be scanned in patients with CSA.

Previous imaging studies investigating the prognostic value of contrast-enhanced MRI for RA development in at-risk individuals used a unilateral scanning protocol, including one foot and one hand, due to clinical feasibility.^2 7 8^ A study by Boer *et al* revealed that the presence of foot inflammation did not occur without the presence of hand inflammation in CSA and did not improve the predictive accuracy of MRI.^7^ Similar findings were observed in patients with undifferentiated arthritis.^9^ Scanning the feet provides no additional value in detecting the presence of subclinical inflammation and can therefore be omitted to reduce costs.

The mDixon MRI sequence has made imaging of two hands feasible;^6^ however, it remains an unresolved question whether one or two hands should be scanned in CSA to detect subclinical inflammation. This is particularly relevant when both hands cannot be scanned simultaneously and imaging both would double the scan time. In patients with established RA, bilateral MRI has been previously explored as a method for assessing disease activity. For accurate assessment of disease activity, the severity of inflammation should be quantified. Studies have shown that scanning two hands yields more optimal results than scanning only one. Additionally, the presence and severity of MRI-detected inflammation was not equally distributed between the hands in patients with classified RA.^10^,^12^ Whether the presence of subclinical inflammation at the at-risk stage of CSA is asymmetrical as well is still unknown.

As subclinical inflammation during the risk stage of CSA is less pronounced than that observed in RA at diagnosis, we hypothesised that its presence will be mostly asymmetrical. If so, this could imply that MRI analysis of two hands may be preferable to optimally detect subclinical inflammation. Hence, aiming to optimise the utility of mDixon MRI in the CSA risk stage, we compared subclinical inflammation detection using data from two hands compared with one. Additionally, we studied the distribution of subclinical inflammation in the hands in CSA.

## Methods

### Patients

All patients studied were consecutively included in the Leiden observational CSA cohort. This inception cohort was set up in 2012 in the Leiden University Medical Center (Netherlands), which is the only referral centre in a healthcare population of >400 000 inhabitants to study the symptomatic phase of RA without clinically detectable arthritis. Inclusion criteria were having arthralgia of the small joints <1 year that was, according to the clinical expertise of the rheumatologist, suspected to progress to RA over time. No further criteria were made with regards to the type of symptoms and thus inclusion was essentially based on the expert opinion of the rheumatologist. Importantly, CSA was not present if clinical arthritis was observed at physical examination or another explanation for the arthralgia was more likely (eg, osteoarthritis or fibromyalgia).

Participants gave written informed consent to participate in the study before taking part. Detailed information on the ongoing CSA cohort and an overview of the variables that are collected can be found elsewhere.^13^

### Self-reported painful joints

Pain data were collected through an online questionnaire with a mannequin diagram. Participants indicated whether they experienced pain in the left or right wrist, metacarpophalangeal (MCP) 1–5 joints, proximal interphalangeal 1–5 joints and distal interphalangeal 2–5 joints. This corresponded to 15 joints per hand, resulting in a total score ranging from 0 to 30.

### MRI

#### MRI acquisition

**Figure 1:**
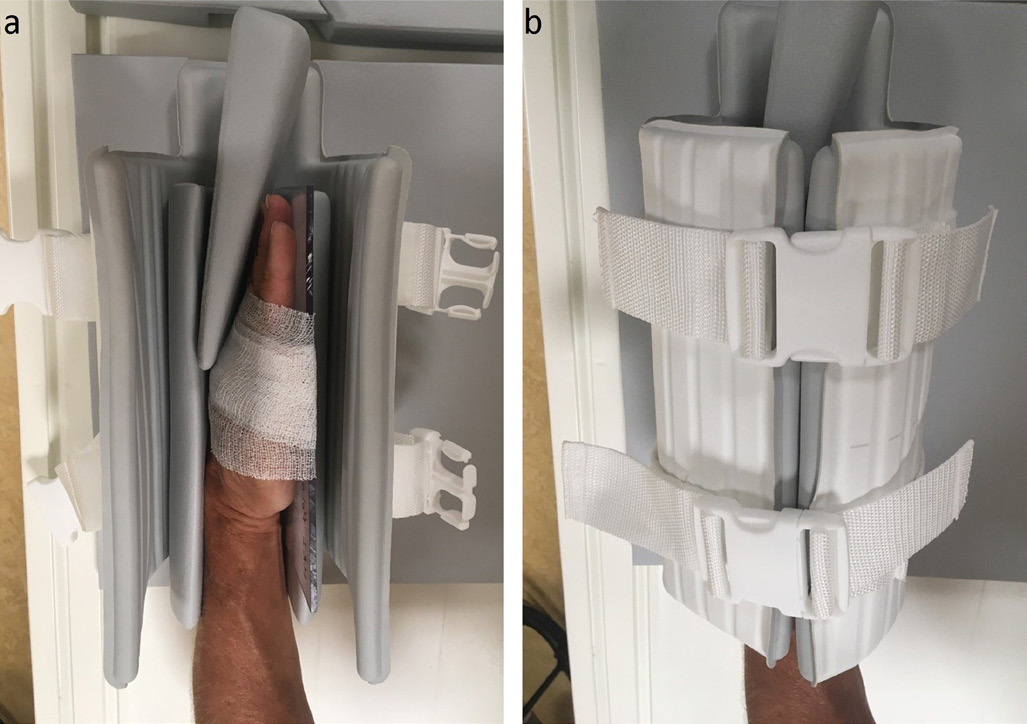
Example of patient hand positioning in a 16-channel coil; because hands are scanned one at a time, scanning two hands doubles the scanning time. Patients were positioned supine with the hand alongside the body, stabilised in a dedicated hand/wrist 16-channel hand/wrist coil using cushions and a glass plate. Separate scans were made of the two hands, each with an acquisition time of 5:20 min. (**a**) Hand positioning within the coil. (**b**) Coil closed to minimise movement artefacts during scanning.

A 3D proton density (PD) mDixon sequence was performed using a 3.0T MRI system (Philips, Best, the Netherlands) generating four images: in-phase, out-of-phase, water-only and fat-only. Coronal and axial reconstructed images were available. MRI scans were performed within 2 weeks of the first clinical presentation and patients were asked not to use non-steroidal anti-inflammatory drugs 24 hours prior to the scan. Both hands were imaged from the wrist to the distal phalanges. Simultaneous scanning of both hands, with the arms in the superman or prayer position, was initially attempted but proved insufficiently robust. Patients found the superman position uncomfortable; the prayer position was insufficiently stable (prone to movement artefacts). It was observed that the optimal position was when patients were placed in the supine position with the hand alongside the body. Consequently, separate scans were made of the two hands (in fact, the separate scanning of the left and right hands fuelled the current study, as it doubled the scanning time). The images from the left and right hands were acquired using a dedicated hand/wrist 16-channel coil, each with an acquisition time of 5:20 min. An example of the hand positioning is presented in figure 1. The following acquisition parameters were used: repetition time (TR) =1300 ms, echo time (TE) =59 ms, turbo spin echo (TSE) factor=47, flip angle (FA) =90⁰, bandwidth (BW) =900.3 Hz, field-of-view (FOV) =250 × 130 mm, matrix=356×186, voxel size=0.7 mm^3^, number of slices=230, oversampling=1. Enhanced gradient mode was enabled, and no uniformity correction was applied.

##### MRI reading

The coronal and axial water-only images were evaluated for the wrist and MCP2–5 joints according to the Outcome Measures in Rheumatology Clinical Trials RA MRI scoring (RAMRIS) system, which is the standard method for manually quantifying subclinical inflammation and structural changes on MRI.^14 15^ All readers were trained with an independent mDixon MRI data set consisting of >400 patients with early arthritis and CSA. Intraobserver and interobserver reliability were calculated before the start of the study. Intraclass correlation coefficients ranged from 0.92 to 0.96. Interclass correlation coefficients ranged from 0.72 to 0.78 for synovitis, 0.78 to 0.80 for tenosynovitis and 0.91 to 0.95 for osteitis. Interobserver reliability will be confirmed in this study. MRIs were scored by two readers blinded to clinical data. Subclinical inflammation was considered present (MRI-positive) if both readers scored grade ≥1 for synovitis, tenosynovitis and/or osteitis (range 0–3) at the same location. Erosions were not included, as previous work has demonstrated that MRI-detected erosions do not provide additional value beyond subclinical inflammation for predicting RA development in CSA.^16^ The thumb was excluded in the analysis, as it was not scored perpendicular to the scan direction. The bones of the first carpometacarpal joint (the base of the first metacarpal and the trapezium) and triscaphoid joint (comprising the scaphoid, trapezium and trapezoid) were also excluded, as these are preferential locations for degenerative changes or locations with MRI signal intensities in the general population.^17 18^ Because signal intensities present at these locations may be non-specific, these locations may not contribute to the comparison between scanning one or two hands to detect subclinical inflammation as a risk factor for RA development.

### IA and RA development

The development of IA was defined as the presence of joint swelling on physical examination by the rheumatologist during follow-up. Among those who developed IA, RA was defined as clinically apparent arthritis with fulfilment of the 1987 and/or 2010 criteria for RA, or clinically apparent arthritis with a clinical diagnosis of RA and start of disease-modifying antirheumatic drug (DMARD) treatment.^19 20^ Protocolised study visits were scheduled at 4 months, 12 months and 24 months with additional visits arranged if patients experienced a worsening of symptoms. Follow-up ended when patients developed IA or after completion of the 2-year follow-up period. During follow-up, patients were not treated with DMARDs, including glucocorticoids.

Since patients are still being followed in the CSA cohort, we required that all patients included in this analysis had at least 6 months of follow-up. These patients were consecutively included between September 2021 and October 2024. IA and RA development was evaluated over the entire available follow-up period for all patients.

### Statistical analysis

The relationship between the development of IA and RA and subclinical inflammation detected by bilateral versus unilateral analysis was compared. For the bilateral analysis, MRI data of both hands were used. For the unilateral analysis, only data obtained from one hand were assessed, that is, the most painful hand based on the highest number of self-reported painful joints at baseline. If each hand had the same number of self-reported painful joints or these data were missing, the dominant hand was selected for the unilateral analysis. If dominance was unknown, the right hand was used. The hazard ratio (HR) for IA and RA development was calculated during the entire available follow-up of all patients. Test characteristics, including area under the receiver operating characteristics curve (AUC), sensitivity and specificity, were assessed at 6 months. Absolute differences in test characteristics between bilateral and unilateral analyses were determined. The category-based Net Reclassification Index (NRI) was calculated to assess the net percentage of patients correctly classified when data from both hands were used instead of one.^21^

Second, the distribution of subclinical inflammation at patient level was examined, distinguishing between bilateral and unilateral joint involvement. Bilateral joint involvement was defined as subclinical inflammation present in ≥1 location in each hand, regardless of whether the affected locations were the same. Unilateral joint involvement was defined as subclinical inflammation limited to only one hand. The distribution of subclinical inflammation (synovitis, tenosynovitis and/or osteitis) between the hands was assessed for all patients, and each inflammatory feature was also evaluated individually. Additionally, subclinical inflammation was analysed separately for patients who progressed to IA (convertors) and those who did not (non-convertors).

Since patients with symmetrical symptoms may also have more symmetrical subclinical inflammation, we evaluated whether unilateral MRI analysis might be sufficient in this subgroup. Therefore, a sensitivity analysis was conducted in patients with CSA with either symmetrical or asymmetrical joint symptoms. Joint symptoms were considered symmetrical when patients with CSA self-reported the same number of painful joints in both hands. An unequal number of self-reported painful joints was considered asymmetrical. For both subgroups, we calculated the HR and test characteristics for predicting IA development using bilateral and unilateral MRI analyses. In addition, the distribution of subclinical inflammation in both subgroups was evaluated.

Inter-reader reliability was evaluated using intraclass correlation coefficients based on a two-way mixed-effects model with absolute agreement and average measures. Intraclass correlation coefficients of <0.50, 0.50–0.75, 0.75–0.90 and >0.90 indicated poor, moderate, good and excellent reliability, respectively.^22^ All analyses were performed using STATA V.18.5.^23^

## Results

### Baseline characteristics

In total, 139 patients with CSA were included and studied. Patients were mostly female (76%), the mean age was 46 years (SD 13 years) and the median symptom duration at first presentation was 18 weeks (IQR 10–31). Anticitrullinated protein antibodies (ACPAs) and rheumatoid factor (RF) positivity were observed in 14% and 19% of patients with CSA, respectively. The median 68 tender joint count (TJC68) on physical examination was 4 (IQR 2–8), while the median self-reported painful hand joint count was 9 (IQR 3–16) (table 1).

**Table 1:** Baseline characteristics of patients with CSA who underwent mDixon MRI

Characteristics	All patients (n=139)
Female sex, n (%)	106 (76)
Age, mean (SD**)**	46 (13)
Symptom duration (weeks), median (IQR**)**	18 (10–31)
Morning stiffness (>60 min), n (%)	51 (37)
Self-reported painful hand joints, median (IQR)*	9 (3–16)
TJC68, median (IQR)	4 (2–8)
CRP positive (≥5 mg/L), n (%)	26 (19)
ACPA or RF positive, n (%)	29 (21)
ACPA positive (≥7 U/mL), n (%)	19 (14)
RF positive (≥3.5 IU/mL), n (%)	26 (19)

* The number of self-reported painful hand joints was collected at baseline through an online questionnaire with a mannequin diagram. Participants indicated whether they experienced pain in the left or right wrist, MCP1–5, PIP1–5 and DIP2–5 joints, allowing for a total range of 0–30 painful joints.

ACPA, anticitrullinated protein antibodies; CRP, C reactive protein; CSA, clinically suspect arthralgia; DIP, distal interphalangeal; MCP, metacarpophalangeal; mDixon MRI, modified Dixon MRI; PIP, proximal interphalangeal; RF, rheumatoid factor; TJC68, tender joint count of 68 joints.

### Reliability between readers

Interobserver reliability from the training data set was confirmed in this cohort. Intraclass correlation coefficients demonstrated good to excellent reliability, with values of 0.97 (95% CI 0.96 to 0.98) for subclinical inflammation, 0.80 (95% CI 0.73 to 0.86) for synovitis, 0.95 (95% CI 0.93 to 0.96) for tenosynovitis and 0.76 (95% CI 0.68 to 0.84) for osteitis.

### Prognostic performance

During the median follow-up of 21 months (IQR 8–25), 16 patients developed IA of whom 13 fulfilled the 1987 and/or 2010 classification criteria for RA. The bilateral mDixon MRI analysis showed a stronger association with IA development compared with the unilateral analysis; HR 4.80 (95% CI 1.09 to 21.11) versus 2.34 (95% CI 0.84 to 6.49), respectively. The difference in HRs between both MRI analyses was even more pronounced for RA development; HR 8.17 (95% CI 1.06 to 62.86) versus 3.23 (95% CI 0.98 to 10.66) (table 2).

**Table 2:** HR and test characteristics of bilateral versus unilateral mDixon MRI analysis for predicting IA and RA development in patients with CSA

mDixon MRI analysis	HR (95% CI)	Sens (95% CI)	Spec (95% CI)	AUC (95% CI)
IA development				
Bilateral hands	4.80 (1.09 to 21.11)	0.83 (0.52 to 0.98)	0.42 (0.33 to 0.51)	0.63 (0.51 to 0.74)
Unilateral hand	2.34 (0.84 to 6.49)	0.58 (0.28 to 0.85)	0.57 (0.48 to 0.66)	0.58 (0.42 to 0.73)
RA development				
Bilateral hands	8.17 (1.06 to 62.86)	0.90 (0.56 to 1.00)	0.42 (0.33 to 0.51)	0.66 (0.55 to 0.77)
Unilateral hand	3.23 (0.98 to 10.66)	0.70 (0.35 to 0.93)	0.57 (0.48 to 0.66)	0.64 (0.48 to 0.79)

For the unilateral MRI analysis the most painful hand was used, which was based on the hand with the highest number of self-reported painful joints. The HR for IA and RA development was calculated over the entire follow-up period. Test characteristics for predicting IA and RA development, including sensitivity, specificity and AUC, were assessed at 6 months. No correction for signal abnormalities in age-matched, symptom-free individuals has yet been applied. Consequently, the results on specificity and AUC should not be interpreted as the final accuracy of mDixon MRI; however this does not affect the comparison between an unilateral or bilateral hand MRI.

AUC, area under the receiver operating characteristics curve; CSA, clinically suspect arthralgia; IA, inflammatory arthritis; mDixon MRI, modified Dixon magnetic resonance imaging; RA, rheumatoid arthritis; Sens, sensitivity; Spec, specificity.

Test characteristics were assessed at 6 months (table 2). For IA development, the bilateral analysis resulted in a higher sensitivity compared with the unilateral analysis (0.83, 95% CI 0.52 to 0.98 vs 0.58, 95% CI 0.28 to 0.85). Thus, with the data obtained from two hands, more patients that progressed to IA were identified at baseline. Specificity, however, was lower for bilateral versus unilateral analysis (table 2). Analysis using bilateral MRI data resulted in a numerically slightly higher AUC value compared with unilateral analysis for IA development (table 2). Absolute differences for the sensitivity, specificity and AUC were +25%, −15% and +5%, respectively. With RA development as the outcome, findings were similar (table 2). Bilateral analysis resulted in a higher sensitivity, lower specificity and an almost similar AUC (table 2), with absolute differences of +20%, −15% and +2%, respectively.

The NRI for IA as outcome was 0.10, indicating that the bilateral analysis increased the proportion of correctly classified patients by 10% (online supplemental table S1A). This was due to the finding that with the data obtained from two hands (compared with one hand) 25% of convertors were correctly reclassified, while 15% of non-convertors were incorrectly reclassified. With RA development as outcome, the NRI was 0.04 (online supplemental table S1B).

### Distribution of subclinical inflammation

**Figure 2:**
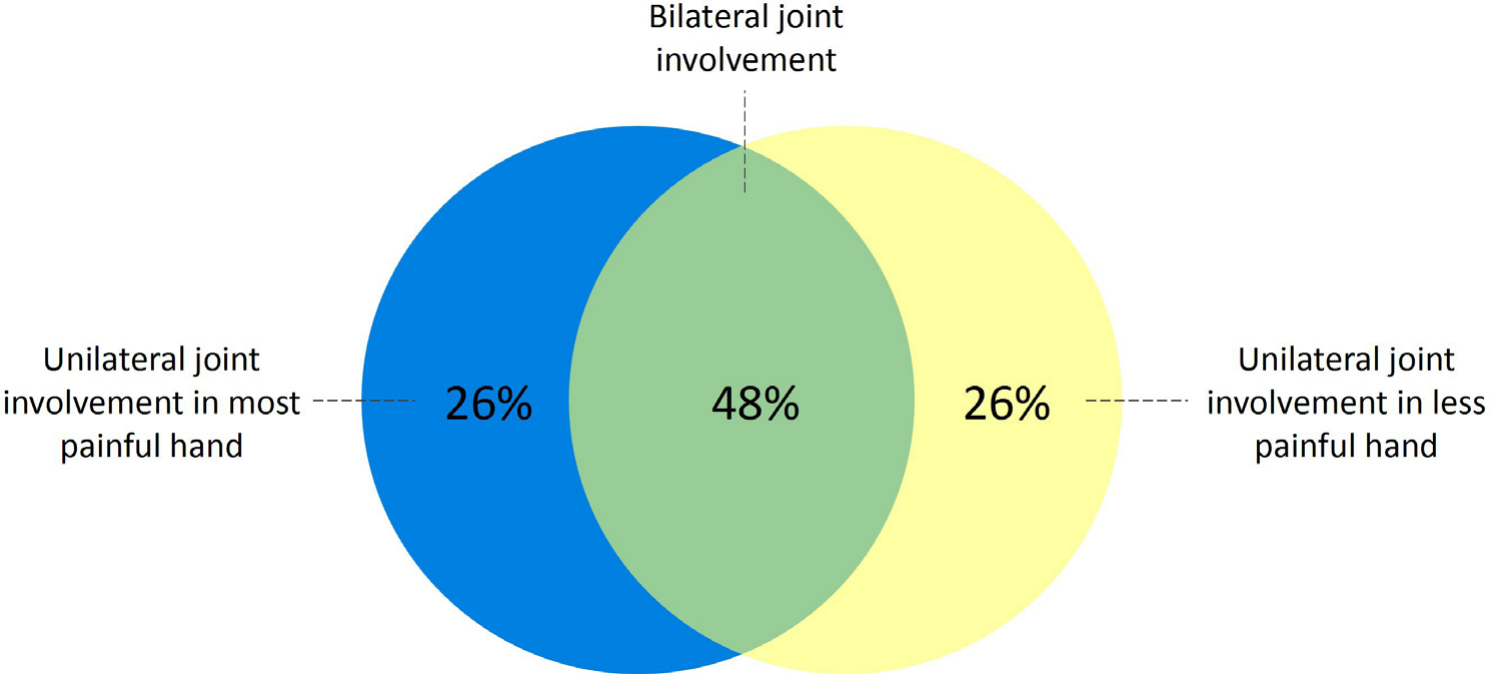
Distribution of subclinical inflammation in patients with CSA with a positive mDixon MRI. 84 out of the 139 patients with CSA had a positive mDixon MRI, defined as the presence of grade ≥1 subclinical inflammation (synovitis, tenosynovitis or osteitis) scored by two readers at the same location. Joint involvement was classified as bilateral if it was present in ≥1 location in each hand, regardless of whether the affected locations were identical. Selection of the most painful hand was based on the highest number of self-reported painful joints. CSA, clinically suspect arthralgia; mDixon MRI, modified Dixon MRI.

**Figure 3:**
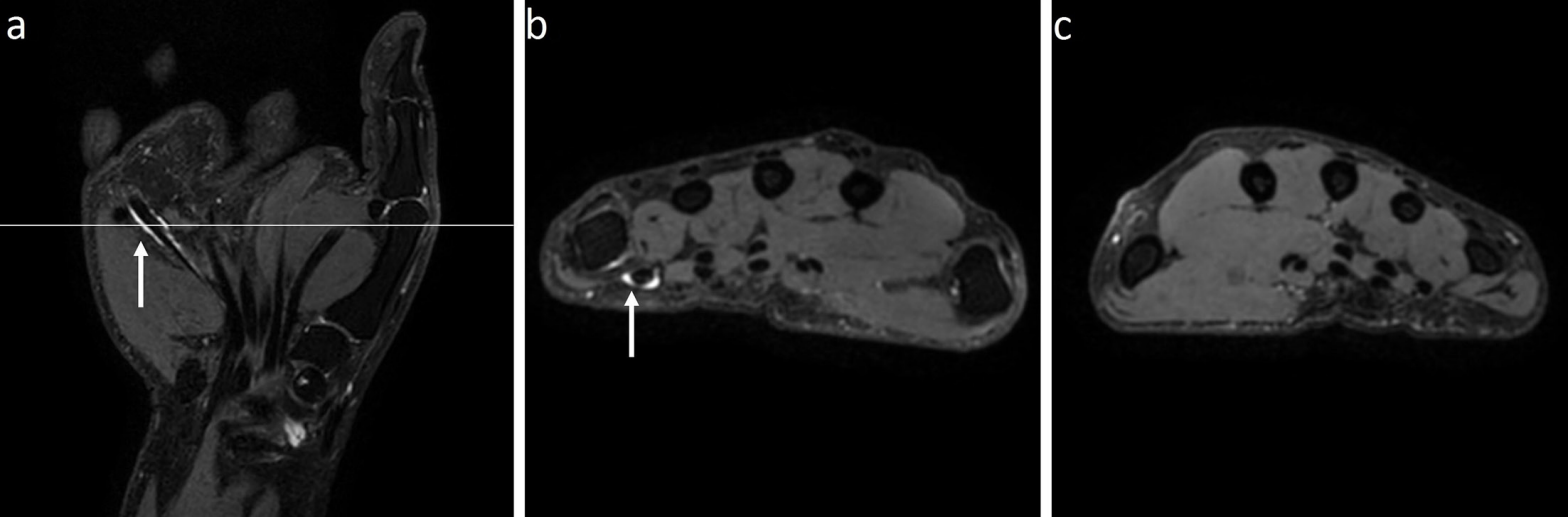
An mDixon MRI example showing unilateral tenosynovitis in a patient with CSA at one location. A 39-year-old woman with unilateral tenosynovitis on water-only mDixon MRI at one location. (**a**) Coronal and (**b**) axial slices of the right hand and (**c**) axial slice of the left hand at the metacarpophalangeal level. Tenosynovitis was scored in the flexor digitorum tendon at the right fifth metacarpophalangeal joint (indicated by the white arrows). CSA, clinically suspect arthralgia; mDixon MRI, modified Dixon MRI.

Among patients with CSA with a positive MRI, 48% had bilateral joint involvement, while 52% had unilateral involvement. Of those with unilateral joint involvement, half had inflammation in their most painful hand, defined as the hand with the highest number of self-reported painful joints (figure 2). Scanning only one hand would therefore result in missing a quarter of patients with subclinical joint inflammation. A similar pattern was observed for tenosynovitis, which was present unilaterally in 59% of patients with CSA with tenosynovitis (figures3^5^). For synovitis and osteitis, asymmetry was even more pronounced, with 71% and 77% unilateral involvement, respectively (figure 5). No difference in the distribution of subclinical inflammation between converters and non-converters was observed (figure 6).

**Figure 4:**
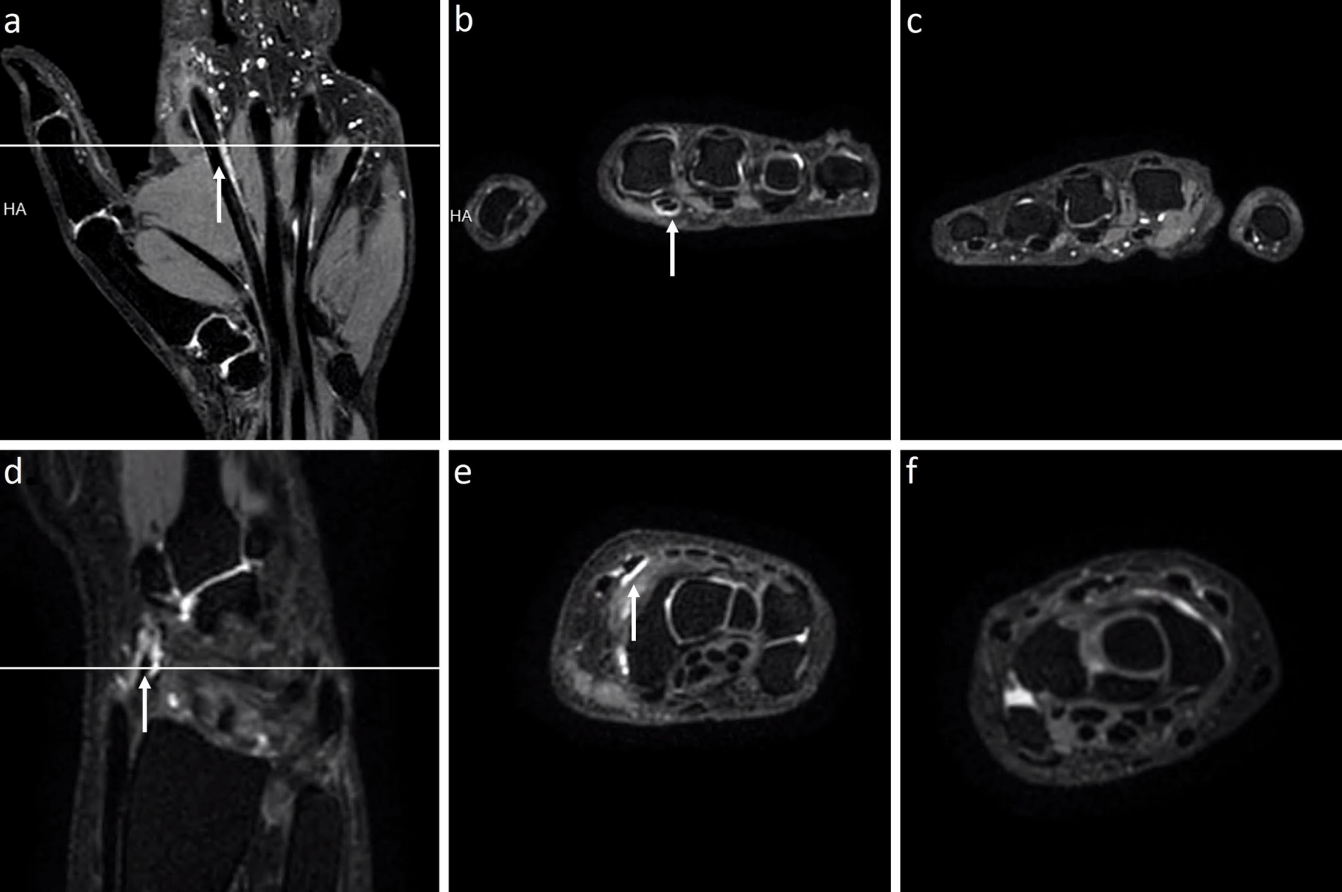
An mDixon MRI example showing unilateral tenosynovitis in a patient with CSA at two locations. A 48-year-old woman with unilateral tenosynovitis on water-only mDixon MRI at two locations. (**a,d**) Coronal and (**b,e**) axial slices of the left hand at two levels. (**c,f**) Axial slices at corresponding levels of the right hand. Tenosynovitis was scored in the flexor digitorum tendon at the left second metacarpophalangeal joint and in the extensor carpi radialis longus and brevis (indicated by the white arrows). A multiloculated ganglion cyst was observed in the right hand, and no subclinical inflammation was present. CSA, clinically suspect arthralgia; mDixon MRI, modified Dixon MRI.

**Figure 5:**
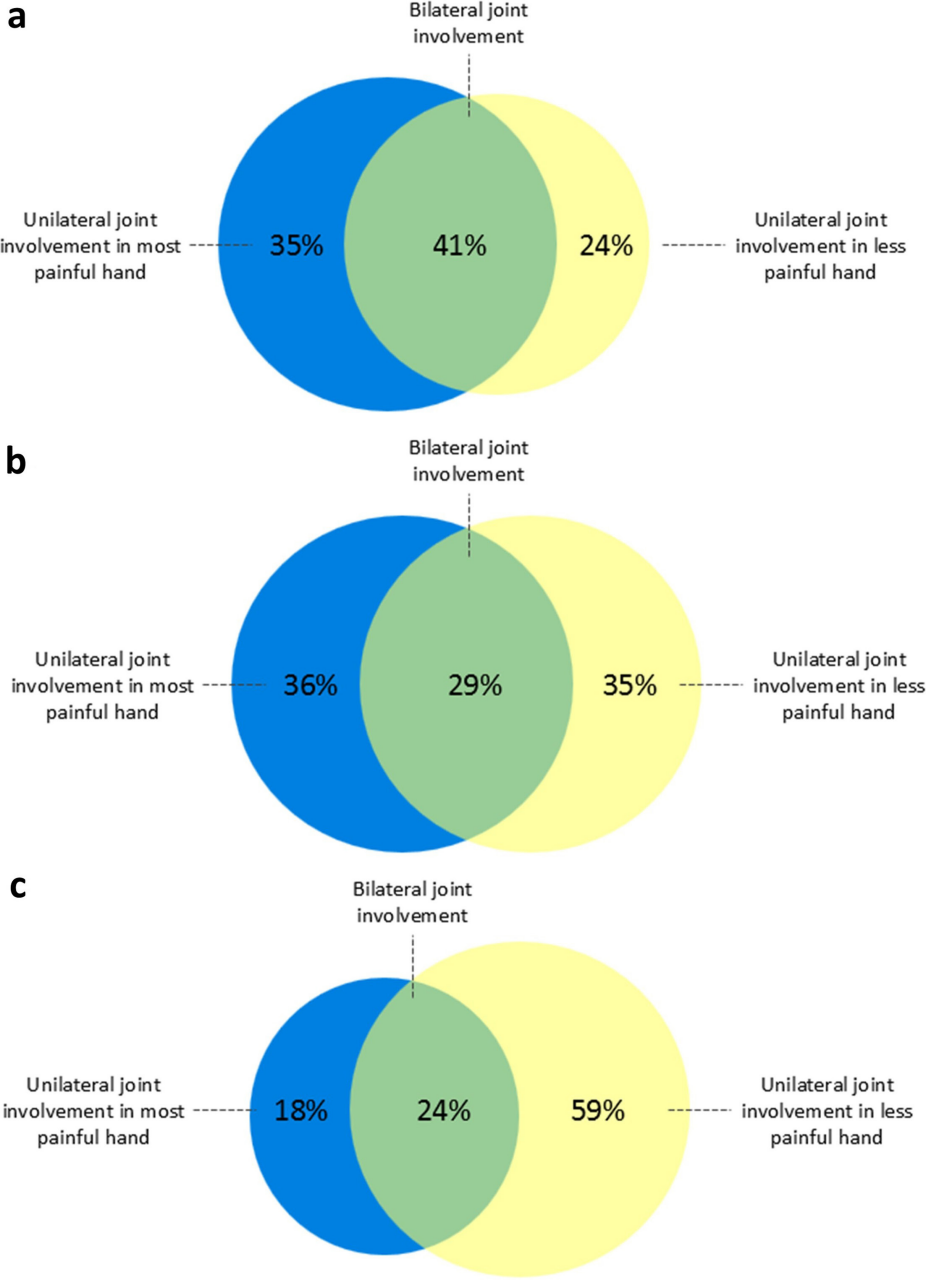
Distribution of subclinical inflammation in patients with CSA with a positive mDixon MRI, stratified by tenosynovitis (**a**), synovitis (**b**) and osteitis (**c**). 84 out of the 139 patients with CSA had a positive mDixon MRI, defined as the presence of grade ≥1 subclinical inflammation (tenosynovitis, synovitis or osteitis) scored by two readers at the same location. Panels **a–c** show mDixon MRI-positive patients with (**a**) tenosynovitis, (**b**) synovitis and (**c**) osteitis. Joint involvement was classified as bilateral if it was present in ≥1 location in each hand, regardless of whether the affected locations were identical. Selection of the most painful hand was based on the highest number of self-reported painful joints. CSA, clinically suspect arthralgia; mDixon MRI, modified Dixon MRI.

**Figure 6:**
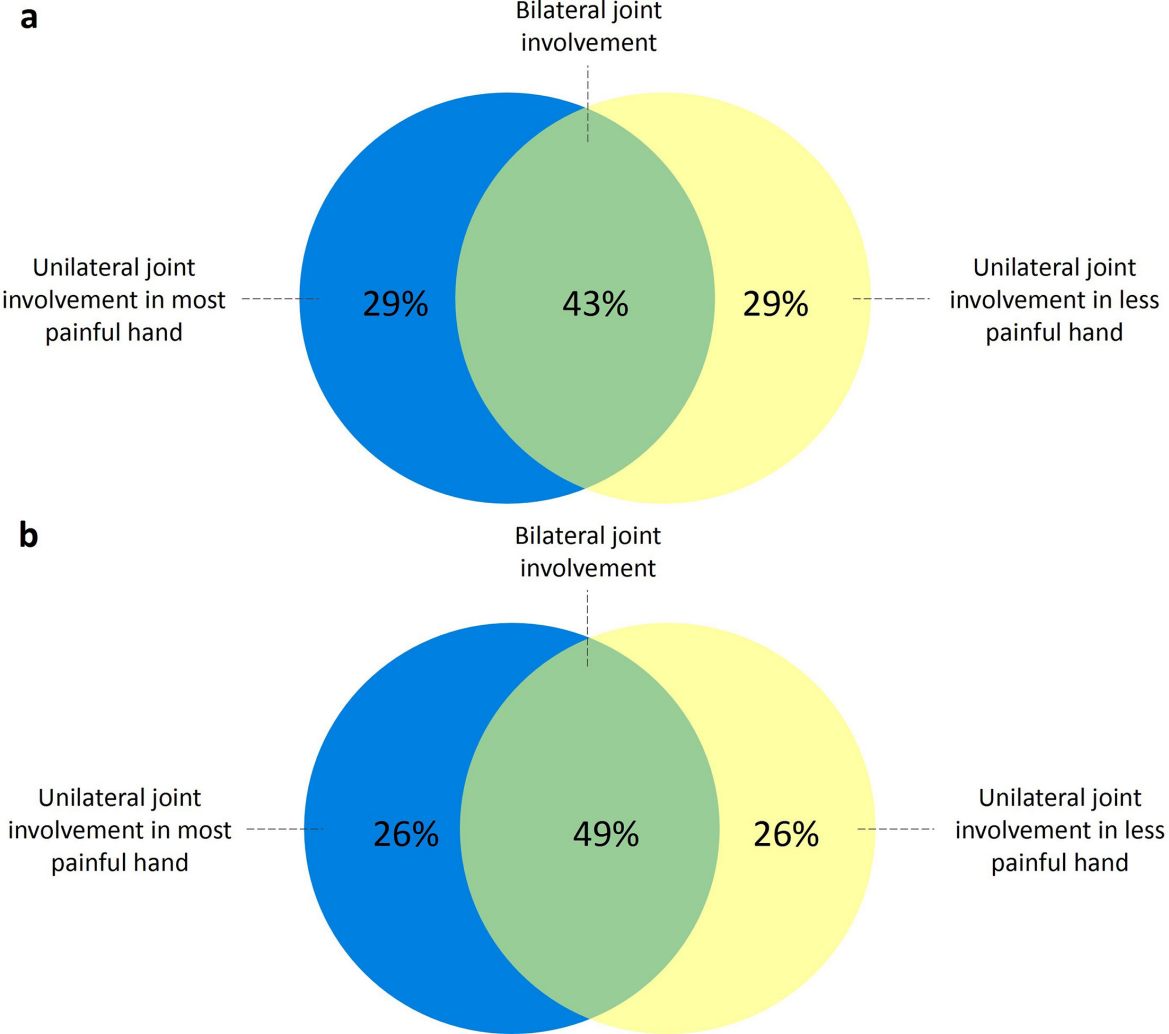
Distribution of subclinical inflammation in patients with CSA with a positive mDixon MRI, stratified for those who developed IA and those who did not. 84 out of the 139 patients with CSA had a positive mDixon MRI and of those patients 14 developed IA during follow-up (**a**), and 70 did not (**b**). MRI positivity was defined as the presence of grade ≥1 subclinical inflammation (synovitis, tenosynovitis or osteitis) scored by two readers at the same location. Joint involvement was classified as bilateral if it was present in ≥1 location in each hand, regardless of whether the affected locations were identical. Selection of the most painful hand was based on the highest number of self-reported painful joints. CSA, clinically suspect arthralgia; IA, inflammatory arthritis; mDixon MRI, modified Dixon MRI.

### Sensitivity analysis

In the stratified analyses of patients with CSA with symmetrical (n=69, 50%) and asymmetrical (n=70, 50%) joint symptoms, results comparable to those of the primary analysis were observed. However, the increase in diagnostic accuracy from bilateral analysis was somewhat greater in patients with asymmetrical symptoms (online supplemental table S2). The distribution of subclinical inflammation in both subgroups was similar to the entire population (online supplemental figure S1).

## Discussion

We compared bilateral versus unilateral mDixon MRI-detected subclinical inflammation as a risk factor for the development of IA and RA in patients with CSA. Although the robust 3D PD mDixon MRI sequence is fast with a scan time of 5 min, due to the scanner and the positioning used in our study, only one hand could be scanned at a time.^6^ If scanning only one hand provides similar information to scanning two hands, the overall scan time could be halved. However, our findings showed that MRI data of two hands resulted in a higher sensitivity for detecting subclinical inflammation and a stronger association with IA and RA development compared with data from one hand. This implies that ideally, two hands should be imaged to detect early subclinical inflammation.^1^

The presence of MRI-detected subclinical inflammation is a known predictor of progression from CSA to RA, which is confirmed in this study. Our results indicate that scanning two hands is optimal to detect subclinical inflammation, explained by the fact that half of the patients with CSA exhibited an asymmetrical distribution of subclinical inflammation. Scanning only one hand could therefore result in missing a quarter of patients with CSA with subclinical inflammation present in the contralateral hand. This finding is in contrast to previous results from the feet, which showed that inflammation in the metatarsophalangeal joints did not occur without concurrent inflammation in the hands.^7^

In this study, subclinical inflammation was defined as grade ≥1 synovitis, tenosynovitis and/or osteitis on MRI of the hands. As such, the presence of subclinical joint inflammation (MRI positivity) was studied. Recently, the EULAR/ACR risk stratification criteria for RA development were developed.^3^ These included only tenosynovitis and showed that the presence of more extensive tenosynovitis corresponded with a higher risk to develop RA. A previous study by Matthijssen *et al* also showed that a greater number of locations with subclinical inflammation is associated with an increased risk of developing RA.^1^ Interestingly, however, the EULAR/ACR risk stratification criteria were developed with MRI data of one hand/foot. To the best of our knowledge, this study is the first evaluating MRI of both hands in CSA. Our finding may indicate that the MRI component of the EULAR/ACR criteria could be improved by scanning both hands instead of just one. However, the added value of MRI compared with clinical and serological variables was presented in these risk stratification criteria. Whether two hands offer added value compared with clinical, serological and unilateral imaging data, and if so, whether this could lead to an improvement in these criteria, remains to be determined. It is also important to note that not all patients with CSA with subclinical inflammation will progress to RA.^2^ Therefore, management or treatment decisions should not be based solely on the presence of MRI-detected inflammation. Instead, the actual predicted risk for RA as provided by the EULAR/ACR risk stratification criteria might be useful to this end.^3^

Bilateral analysis improved reclassification by 10% by detecting more subclinical inflammation. This reflects the improvement of assessing two hands instead of one, not the added value of mDixon MRI itself. We therefore consider the modest improvement observed to be clinically relevant, particularly because a quarter of converters were correctly reclassified.

This is one of the first studies on mDixon MRI in CSA. There are still several issues that need to be resolved before the final value and accuracy of mDixon can be demonstrated. First, the specificity requires further evaluation. From contrast-enhanced MRI, it is known that inflammation-like features can occur in the general population and that these need to be considered to prevent false-positive findings and achieve a high specificity.^17 24^ In our study, we excluded certain locations that might be non-specific and reflect normal variations. These, therefore, may not contribute to the comparison between bilateral and unilateral analysis. Importantly, however, the normal variations, as depicted by mDixon MRI, are not yet known. An extensive study to determine the frequency of signal abnormalities at specific locations in age-matched symptom-free individuals is still ongoing. These data are required to determine the accuracy and specificity in particular. Therefore, the specificity values presented here may not reflect the final specificity of mDixon MRI. As shown before with conventional contrast-enhanced MRI, corrections for normal variants may reduce the number of non-specific findings and thereby improve the specificity, without affecting the sensitivity.^24^ However, this correction will be applied to both hands and may not affect our comparison between bilateral and unilateral analyses. In other words, although the prognostic value and specificities may change, the conclusion about scanning both hands may remain similar as the asymmetrical nature of subclinical joint inflammation in CSA may not change by correcting for normal variants.

Another limitation of our data is that the follow-up duration is relatively short (≥6 months). This explains the fact that we here observed a lower rate of RA development compared with previous reports with a longer follow-up.^25^ Here too, a longer follow-up is needed to determine the final accuracy of mDixon MRI. However, a longer follow-up is also unlikely to affect the conclusion on whether evaluation of one or two hands is optimal.

Interestingly, assessing the most painful hand only was suboptimal. It should be noted that there are different ways to define the most painful hand. In our data, this was defined as the hand with the highest number of painful joints reported by the patient, as indicated on a drawing (mannequin) of the hands, and if these were undetermined, the dominant hand. Although one might expect more subclinical inflammation in the dominant hand due to degenerative changes, this is not supported by evidence, as prior research has shown that in 47% of cases the dominant hand actually had lower RAMRIS scores than the non-dominant hand.^11^ Therefore, we consider it unlikely that selecting the dominant hand biased our results. It is possible that the side the patient experiences as most painful does not always correspond with the side a rheumatologist would identify as the most painful, for example, when physical examination would also be included in this evaluation. We did not investigate this, and therefore this might be a subject for future research. From the current data, we can conclude that when the patient is scanned only on the most painful side (according to the patient), subclinical inflammation is missed.

A consideration for implementing mDixon MRI in routine rheumatology practice is the variation between scanners and scanner vendors. In our set-up (using a Philips scanner, and to allow patients to lie comfortably and to obtain good image quality), each hand had to be scanned separately. For scanners of other vendors, it may be possible to achieve an alternative hand positioning, enabling simultaneous scanning of two hands.^6^ Therefore, scan times and acquisition protocols can vary between different MRI vendors. While optimisation should be performed per vendor, this need not hinder the generalisation of the MRI’s value.

A strength of this study is the consistency of findings for both IA and RA development. The improved prognostic performance was even more evident when assessing RA development compared with IA. Additionally, we studied the symmetry of MRI-detected subclinical inflammation in CSA. It might be expected that patients with CSA with symmetrical joint symptoms would also have symmetrical subclinical inflammation, suggesting limited benefit of bilateral hand analysis. However, our sensitivity analysis showed similar results in patients with CSA with either symmetrical or asymmetrical joint symptoms. This supports the use of bilateral analysis in all patients with CSA, regardless of the (a)symmetry of symptoms.

In conclusion, bilateral mDixon MRI of the hands is preferable to unilateral mDixon MRI for detecting subclinical inflammation in CSA, because of its asymmetrical distribution. Although further work is needed to determine the final accuracy, mDixon MRI holds great promise for rheumatology practice due to its robustness, short scan time and lack of contrast agent requirement.

## Supplementary material

10.1136/rmdopen-2025-006621 online supplemental file 1

## Data Availability

Data are available upon reasonable request.
