# Supplementary material for: Modified Dixon MRI to detect subclinical inflammation in clinically suspect arthralgia as a risk factor for rheumatoid arthritis development: should we image one or two hands?
Source: RMD Open. 2026 Mar 4;12(1):e006621. doi: 10.1136/rmdopen-2025-006621 (PMC12970117; doi:10.1136/rmdopen-2025-006621)
Supplement: online supplemental file 1 [file rmdopen-12-1-s001.docx]

**SUPPLEMENTARY FILES**

**Supplementary Table S1.** Net reclassification index of bilateral versus unilateral mDixon MRI analysis for predicting IA and RA development in CSA patients

**Supplementary Table S2.** Hazard ratio and test characteristics of bilateral versus unilateral mDixon MRI analysis for predicting IA development, stratified for CSA patients with symmetrical and asymmetrical symptoms of the joints

**Supplementary Figure S1.** Distribution of subclinical inflammation in CSA patients with symmetrical symptoms and asymmetrical symptoms of the joints

**Supplementary Table S1. Net reclassification index of bilateral versus unilateral mDixon MRI analysis for predicting IA and RA development in CSA patients**

1. **Inflammatory arthritis**

| **mDixon MRI analysis** | **No IA** | **IA** | **Total** |
| --- | --- | --- | --- |
| **Bilateral hands** |  |  |  |
| Negative MRI | 53 | 2 | 55 |
| Positive MRI | **74** | **10** | 84 |
| **Unilateral hand** |  |  |  |
| Negative MRI | 72 | 5 | 77 |
| Positive MRI | **55** | **7** | 62 |
| **Total** | 127 | 12 | 139 |

1. **Rheumatoid arthritis**

| **mDixon MRI analysis** | **No RA** | **RA** | **Total** |
| --- | --- | --- | --- |
| **Bilateral hands** |  |  |  |
| Negative MRI | 54 | 1 | 52 |
| Positive MRI | **75** | **9** | 87 |
| **Unilateral hand** |  |  |  |
| Negative MRI | 74 | 3 | 76 |
| Positive MRI | **55** | **7** | 63 |
| **Total** | 127 | 10 | 139 |

For the unilateral analysis the most painful hand was used, which was based on the hand with the highest number of self-reported painful joints. A positive mDixon MRI was defined as the presence of grade ≥1 subclinical inflammation (synovitis, tenosynovitis, or osteitis) scored by two readers at the same location. IA and RA development was evaluated over the entire follow-up period for all patients.

*Abbreviations:* *CSA, clinically suspect arthralgia; IA, inflammatory arthritis; mDixon MRI, modified Dixon magnetic resonance imaging; and RA, rheumatoid arthritis*

**Supplementary Table S2. Hazard ratio and test characteristics of** **bilateral versus unilateral mDixon MRI analysis for predicting IA development, stratified for CSA patients with symmetrical and asymmetrical symptoms of the joints**

| **Subgroup** | **mDixon MRI analysis** | **HR (95% CI)** | **Sens (95% CI)** | **Spec (95% CI)** | **AUC (95% CI)** |
| --- | --- | --- | --- | --- | --- |
| Patients with symmetrical symptoms | - Bilateral hands - Unilateral hand | 3.72 (0.43 – 31.82)  2.83 (0.52 – 15.47) | 0.80 (0.28 – 1.00)  0.60 (0.15 – 0.95) | 0.44 (0.31 – 0.57)  0.59 (0.46 – 0.72) | 0.62 (0.41 – 0.82)  0.60 (0.35 – 0.84) |
| Patients with asymmetrical symptoms | - Bilateral hands - Unilateral hand | 5.04 (0.63 – 40.31)  1.75 (0.49 – 6.21) | 0.86 (0.42 – 1.00)  0.57 (0.18 – 0.90) | 0.40 (0.28 – 0.53)  0.54 (0.41 – 0.67) | 0.63 (0.47 – 0.78)  0.56 (0.53 – 0.76) |

Symptoms were considered symmetrical when CSA patients self-reported the same number of painful joints in both hands (n=69). An unequal number was considered asymmetrical (n=70). For the unilateral analysis the most painful hand was used, which was based on the hand with the highest number of self-reported painful joints. The HR for IA development was calculated over the entire follow-up period. Test characteristics for predicting IA development, including sensitivity, specificity, and AUC, were assessed at 6 months. No correction for signal abnormalities in age‑matched, symptom‑free individuals has yet been applied. Consequently, the results on specificity and AUC should not be interpreted as the final accuracy of mDixon MRI; however this does not affect the comparison between an unilateral or bilateral hand mDixon MRI.

*Abbreviations: AUC, area under the receiver operating characteristic curve; CI, confidence interval; CSA, clinically suspect arthralgia; HR, hazard ratio; IA, inflammatory arthritis; mDixon MRI, modified Dixon magnetic resonance imaging; sens, sensitivity; and spec, specificity.*

**Supplementary Figure S1. Distribution of subclinical inflammation in CSA patients with a positive mDixon MRI, stratified for those with symmetrical symptoms and asymmetrical symptoms of the joints**

**
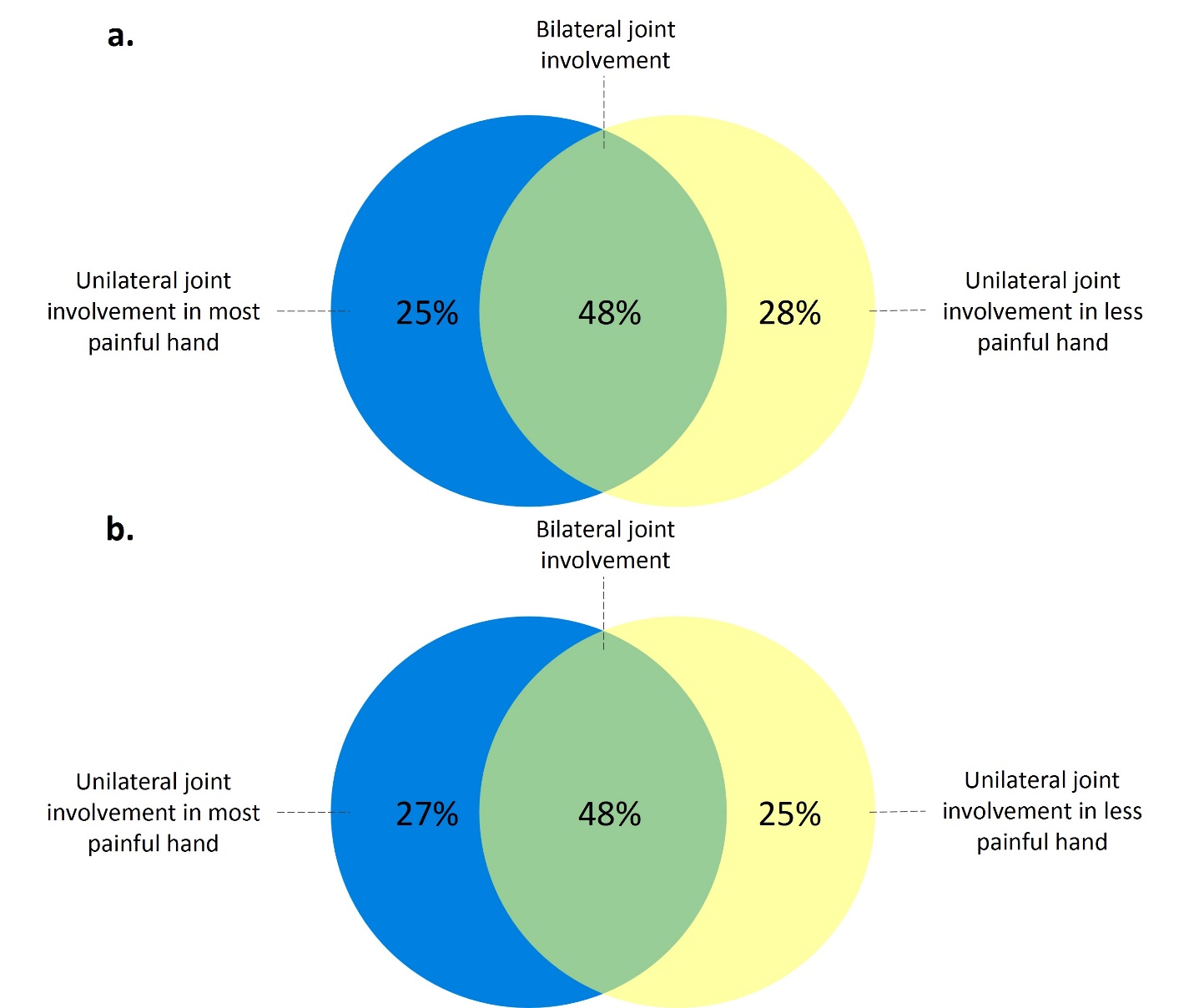
**

84 out of the 139 CSA patients had a positive mDixon MRI, defined as the presence of grade ≥1 subclinical inflammation (synovitis, tenosynovitis, or osteitis) scored by two readers at the same location. (a) mDixon MRI-positive patients with symmetrical and (b) asymmetrical symptoms of the joints. Symptoms were considered symmetrical when patients self-reported the same number of painful hand joints in both hands (n=40). In these patients, the dominant hand (or the right hand if unknown) was selected for the most painful side. Asymmetrical symptoms were defined as an unequal number of painful hand joints between the two hands (n=44). Selection of the most painful hand was based on the highest number of self-reported painful joints. Joint involvement was classified as bilateral if it was present in ≥1 location in each hand, regardless of whether the affected locations were identical.

*Abbreviations: CSA, clinically suspect arthralgia; and mDixon MRI, modified Dixon magnetic resonance imaging.*
